# Reaching for the off switch in nucleolar dominance

**DOI:** 10.1111/tpj.16318

**Published:** 2023-06-02

**Authors:** Craig S. Pikaard, Chinmayi Chandrasekhara, Anastasia McKinlay, Ramya Enganti, Dalen Fultz

**Affiliations:** 1 Department of Biology, Indiana University, Bloomington, IN, USA; 2 Department of Molecular and Cellular Biochemistry, Indiana University, Bloomington, IN, USA; 3 Howard Hughes Medical Institute, Indiana University, Bloomington, IN, USA

**Keywords:** rRNA gene, ribosomal RNA, nucleolus, nucleolus organizer region, transcriptional silencing, chromatin, histone modification, DNA methylation, *Arabidopsis thaliana*, *Arabidopsis suecica*, *Brassica* allopolyploids

## Abstract

Nucleolus organizer regions (NORs) are eukaryotic chromosomal loci where ribosomal RNA (rRNA) genes are clustered, typically in hundreds, to thousands, of copies. Transcription of these rRNA genes by RNA Polymerase I and processing of their transcripts results in the formation of the nucleolus, the sub-nuclear domain in which ribosomes are assembled. Approximately 90 years ago, cytogenetic observations revealed that NORs inherited from the different parents of an interspecific hybrid sometimes differ in morphology at metaphase. Fifty years ago, those chromosomal differences were found to correlate with differences in rRNA gene transcription and the phenomenon became known as nucleolar dominance. Studies of the past 30 years have revealed that nucleolar dominance results from selective rRNA gene silencing, involving repressive chromatin modifications, and occurs in pure species as well as hybrids. Recent evidence also indicates that silencing depends on the NOR in which a rRNA gene is located, and not on the gene’s sequence. In this perspective, we discuss how our thinking about nucleolar dominance has shifted over time from the kilobase scale of individual genes to the megabase scale of NORs and chromosomes, and questions that remain unanswered in the search for a genetic and biochemical understanding of the off switch.

## The intertwined histories of NORs, nucleolar dominance and rRNA genes

**Figure 1.**
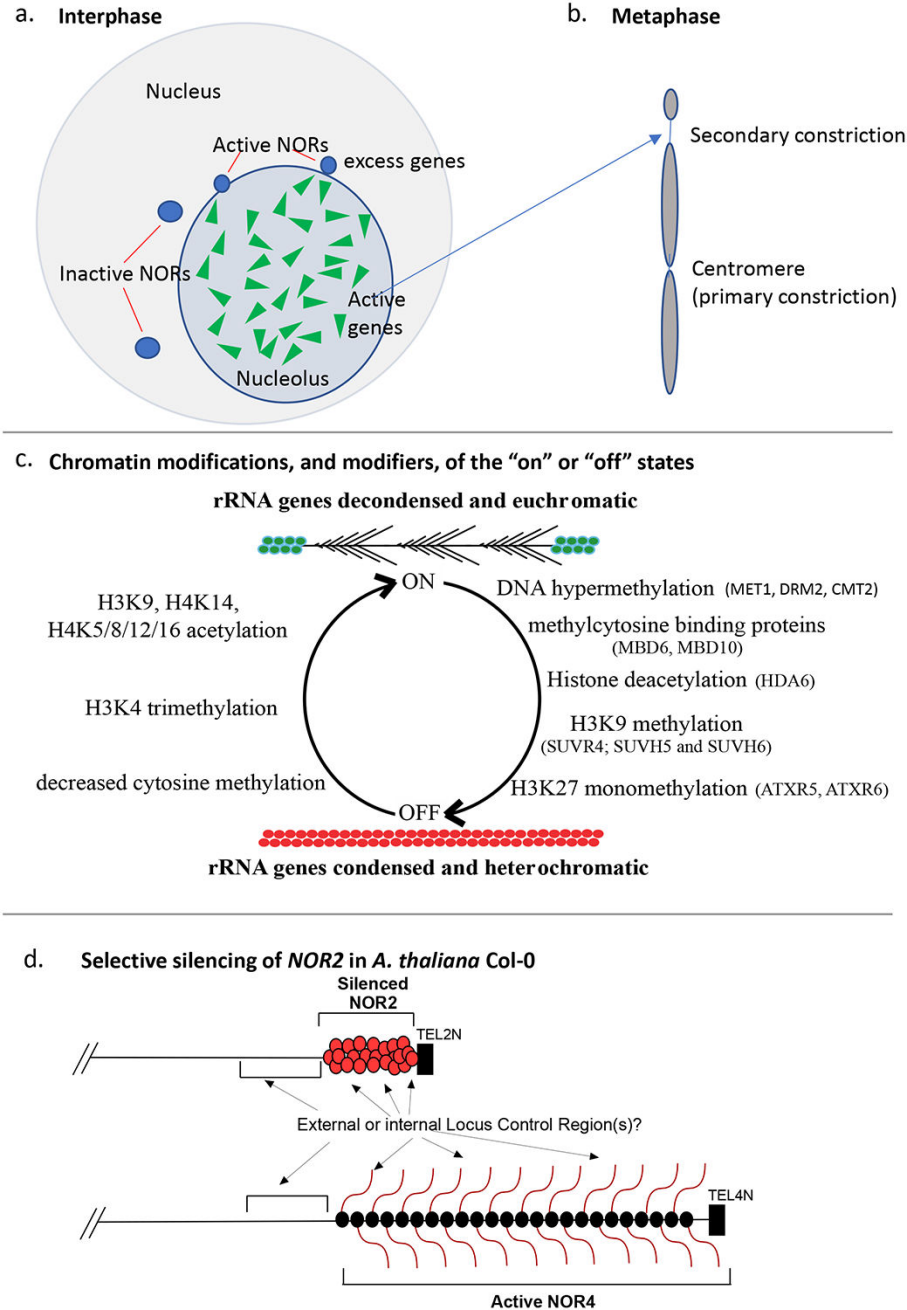
Nucleoli, NORs and rRNA gene expression. a. Nucleoli form within nuclei as a consequence of rRNA gene transcription and processing. Thus, active rRNA genes (green triangles) are located within the nucleolus whereas inactive rRNA genes are located outside the nucleolus, as confirmed by flow cytometry experiments (Pontvianne et al., 2013). The cartoon depicts a diploid nucleus, at interphase, that has nucleoli on two different chromosomes (as in *Arabidopsis thaliana)*, with the NORs identified by DNA-FISH (fluorescence in situ hybridization) using an rRNA gene probe. Inactive genes co-localize within highly condensed foci, and this includes the excess genes of active NORs. b. At metaphase, secondary constrictions are observed at NOR intervals where rRNA genes were transcriptionally active during the prior interphase. c. Chromatin modifications that correlate with the on and off states of plant rRNA genes, and names of some of the Arabidopsis chromatin modifying enzymes (in parentheses) whose mutation or knockdown disrupts rRNA gene silencing (Lawrence and Pikaard, 2004, Probst et al., 2004, Earley et al., 2006, Preuss et al., 2008, Pontvianne et al., 2012, Mohannath et al., 2023). Note that DNA methyltransferase MET1 primarily catalyzes maintenance methylation in the CG context, but also affects CHG maintenance methylation to some extent. CMT2 primarily catalyzes maintenance methylation in the CHH context. DRM2 catalyzes de novo methylation in all contexts (CG, CHG and CHH). d. rRNA genes that map to *NOR2* are selectively silenced whereas genes that map to *NOR4* are expressed in the *A. thaliana* accession, Col-0. The molecular signals and targets that initiate silencing remain undefined but may involve sequences internal to, or flanking, *NOR2* and possibly *NOR4*.

In 1934, McClintock described in maize a chromosomal locus that she named the nucleolar organizer because of its causal association with the nucleolus, the most prominent feature of the nucleus (McClintock, 1934). She recognized that the locus included redundant genetic information because when the locus was split in two by a chromosome break and the resulting chromosome fragments were joined to the two fragments of a different broken chromosome, two nucleoli now formed at the recombinant chromosome junctions (McClintock, 1934). Three decades later, ribosomal RNA genes were found to account for a major class of repetitive DNA in Drosophila and Xenopus and cluster together at NORs, as was also the case in maize (Ritossa and Spiegelman, 1965, Wallace and Birnstiel, 1966, Phillips et al., 1971), providing a molecular explanation for the genetic redundancy deduced by McClintock. Important cytological observations of plant NORs were also made by McClintock’s contemporaries, Heitz and Navashin. Heitz noted that thin strands of chromatin, called secondary constrictions, were observed at metaphase at or near the NORs (Heitz, 1931) and Navashin noted that these secondary constrictions, which were characteristics of specific chromosomes, sometimes failed to form on the chromosome of one parent of an interspecific hybrid (Navashin, 1934). Today, we have molecular biological explanations for these pioneering cytological observations. Transcription of rRNA genes is the function of RNA polymerase I (Pol I) (Roeder and Rutter, 1969, Roeder and Rutter, 1970), resulting in 35-48S pre-ribosomal RNA transcripts (the size varies with species) that are then processed into 18S, 5.8S and 25–28S rRNAs (Turowski and Tollervey, 2015). These rRNAs, together with a fourth rRNA synthesized by RNA polymerase III (5S rRNA), comprise the catalytic core of ribosomes, the protein synthesizing machines of cells. The transcription, processing and post-transcriptional modification of pre-rRNAs, and their assembly with ~80 ribosomal proteins, involves hundreds of proteins and small RNAs (Pederson, 2011, Grummt and Langst, 2013, Sharifi and Bierhoff, 2018, Saez-Vasquez and Delseny, 2019). Collectively, these activities account for formation of the nucleolus as an RNA and protein-rich entity with properties of a phase-separated biological condensate (Brangwynne et al., 2011, Feric et al., 2016, Hori et al., 2023)(see Figure 1a). The persistent binding of Pol I transcription factors to active rRNA genes inhibits their condensation at metaphase (McStay and Grummt, 2008, Prieto and McStay, 2008), accounting for the secondary constrictions described by Heitz (Figure 1b). The formation of secondary constrictions at NORs of only one parent of an interspecific hybrid, as described by Navashin, reflects the transcription of the rRNA genes inherited from only one parent, as demonstrated by Honjo and Reeder (Honjo and Reeder, 1973), who gave us the term “nucleolar dominance” (suggested review articles from different decades include (Reeder, 1985, Pikaard, 1999, McStay, 2006, Borowska-Zuchowska et al., 2023)).

## rRNA genes are subject to developmental and metabolic control

Ribosomal RNA genes are subject to both positive and negative regulation. Positive control mechanisms impact Pol I transcription initiation, elongation and termination, and have been studied most extensively in mammals, Xenopus and yeast (Moss et al., 2007, Goodfellow and Zomerdijk, 2013, Sharifi and Bierhoff, 2018). A generalization gleaned from decades of study is that growth signals that positively regulate rRNA synthesis do so through post-translational modification, such as phosphorylation of Pol I and/or its general transcription factors (Moss et al., 2007, Hori et al., 2023). This allows the rate of Pol I initiation and rRNA synthesis to be tuned to the cellular demand for ribosomes by altering the RNA output from genes already engaged in transcription.

Ribosomal RNA genes are also subject to negative control, which has been studied in diverse eukaryotes including plants, mammalian cell lines, budding yeast and Drosophila (Grummt and Pikaard, 2003, McStay and Grummt, 2008). Collectively, these studies have revealed that rRNA genes can be silenced by mechanisms involving the establishment and maintenance of repressive chromatin modifications. Early evidence came from cytogenetic studies of allopolyploid cereals in which treatment with a chemical inhibitor of DNA methylation disrupted progenitor-specific NOR inactivity, as measured by NOR silver staining (Viera et al., 1990a, Castilho et al., 1995, Neves et al., 1995, Silva et al., 1995). However, it seemed clear that DNA methylation could not fully explain nucleolar dominance because Drosophila hybrids display nucleolar dominance (Durica and Krider, 1977, Durica and Krider, 1978) but do not methylate their DNA. This inspired experiments in which we showed that nucleolar dominance in Brassica allotetraploids could be disrupted using either chemical inhibitors of histone deacetylation or chemical inhibitors of DNA methylation (Chen and Pikaard, 1997a). Moreover, we found that treatment with both inhibitors at the same time did not result in additive or synergistic effects, suggesting that DNA methylation and histone deacetylation are aspects of the same repression pathway. Evidence supporting this hypothesis came from an assay in which we performed chromatin immunoprecipitation (ChIP) using antibodies against Pol I, or specific histone modifications, and then tested the methylation status of the immunoprecipitated DNA using methylation-sensitive DNA endonucleases followed by PCR amplification, an assay we named ChIP-chop-PCR (Lawrence et al., 2004). These studies, conducted using the allotetraploid hybrid species *Arabidopsis suecica*, showed that DNA methylation and histone modification states in the vicinity of rRNA gene promoters change in concert with one another when genes are in the “on” or “off” states (Lawrence et al., 2004). Meanwhile, studies of cultured mouse cells identified a protein complex that recruits DNA methyltransferase and histone deacetylase activities to bring about the silencing of ~50% of the total rRNA gene pool (Santoro et al., 2002). Likewise, studies in yeast showed that the histone deacetylase Rpd3 is involved in rRNA gene silencing as cultures reach stationary phase (Sandmeier et al., 2002). Collectively, these studies showed that rRNA gene silencing in plants, mammals and yeast all involve similar repressive histone modifications. In plants and animals (but not yeast) these histone modifications are further coordinated with changes in DNA methylation. The studies also indicated that nucleolar dominance in genetic hybrids and selective rRNA gene silencing in non-hybrids are manifestations of the same mechanism(s), whose purpose is to limit the total number of rRNA genes that is active (Grummt and Pikaard, 2003, McStay, 2006). Importantly, selective rRNA gene silencing is not permanent. Instead, studies in plants, Drosophila, and Xenopus have shown that nucleolar dominance is developmentally regulated (Castilho et al., 1995, Neves et al., 1995, Chen and Pikaard, 1997b, Pontes et al., 2007) (Earley et al., 2010) (Greil and Ahmad, 2012) (Maciak et al., 2016, Warsinger-Pepe et al., 2020). It may be that species only need all (or most) of their rRNA genes at one or more specific periods of their life cycles, such as embryogenesis or other periods of rapid cell proliferation, but shut down the excess genes at other times of development.

Collectively, the known mechanisms of rRNA gene up-regulation and down-regulation suggest that control occurs on two major levels, with selective silencing used as a form of coarse control to limit the number of genes that is available for transcription, and post-translational modification of transcription factors then fine-tuning the transcriptional output of these active genes.

## How are rRNA genes chosen for selective silencing?

The hundreds of rRNA genes within the nucleus of most pure species (non-hybrids/allopolyploids) are extremely similar in sequence, which is why NORs are among the most difficult genomic loci to assemble from DNA sequencing data. So how can rRNA genes be discriminated from one another to allow some to be “on” and others to be “off”? An initial hypothesis (Reeder, 1985), put forward based on transient expression studies of rRNA minigenes injected into Xenopus oocytes in ~1000-fold excess over the endogenous genes, suggested that the dominant set of rRNA genes have a higher number of repeats that function as enhancer elements within their intergenic spacers, thereby sequestering one or more limiting transcription factors. However, numerous subsequent observations and direct tests have failed to support this hypothesis. For instance, in three Brassica allotetraploids, representing different combinations of three progenitor diploid genomes, we showed that there is no correlation between intergenic spacer length or spacer repeat number and nucleolar dominance (Chen and Pikaard, 1997b). Moreover, nucleolar dominance occurs in marine copepods whose rRNA gene intergenic spacers lack repeated sequences (Flowers and Burton, 2006). The idea that dominant rRNA genes have a higher binding affinity for transcription factors is also incompatible with our demonstration that in *Arabidopsis thaliana* x *A. arenosa* hybrids bred to have 1:3, 2:2 or 3:1 genome doses from the two progenitors, the direction of nucleolar dominance could be reversed as the genome dosage changed (Chen et al., 1998). If one progenitor’s rRNA genes have a higher binding affinity for a limiting transcription factor, based on their sequence or enhancer content, these genes should always compete best for that factor and be transcribed, even if outnumbered. Likewise, using a Brassica *in vitro* Pol I transcription system we developed (Saez-Vasquez and Pikaard, 1997) we tested whether dominant rRNA gene promoters would outcompete underdominant promoters as the DNA concentration was raised to very high levels in order to cause transcription factors to become limiting relative to the number of gene promoters in the reaction. However, we found no difference in the competitive strength of the promoters at any DNA concentration (Frieman et al., 1999). The demonstration that underdominant rRNA genes are expressed upon chemical or genetic interference with DNA methylation or histone modification is yet another indication that transcription factors are not limiting or sequestered by the dominant set of genes. Instead, the evidence suggests that silent genes are merely denied access to these transcription factors.

As discussed above, multiple lines of evidence suggest that something other than transcription factor concentrations, binding affinities, or binding site (e.g. enhancer) numbers account for nucleolar dominance. Thus, upon examining a new model species and finding that dominant and under-dominant rRNA genes differ in structure, it is prudent to refrain from concluding that these structural differences are causative with respect to nucleolar dominance. The genes that differ in structure are also genetically linked, for millions of basepairs, to adjacent chromosomal sequences, and it could be that these distant sequences are key to nucleolar dominance.

Instead of dominant rRNA genes being selectively activated, for which there is scant evidence, there is ample evidence that underdominant genes are selectively silenced, with numerous chromatin modifying activities important for rRNA gene silencing having been identified in *Arabidopsis thaliana* or *Arabidopsis suecica* allotetraploids (Figure 1C). However, identification of these chromatin modifying activities in plants, many of which have homologs that play corresponding roles in nucleolar dominance in mammals (Grummt and Pikaard, 2003), has failed to explain how specific rRNA genes are singled out for silencing. The finding that noncoding RNAs play roles in rRNA gene silencing in mouse cells (Mayer et al., 2006, Santoro et al., 2010) and in nucleolar dominance in *Arabidopsis suecica* (Preuss et al., 2008) thus generated some excitement. In the case of *A. suecica*, the rRNA genes inherited from *A. thaliana* and *A. arenosa* differ sufficiently in sequence that their discrimination by species-specific noncoding RNAs is feasible. However, in pure species, such as *A. thaliana*, in which specific rRNA genes are also silenced, the promoter regions of the many hundreds of rRNA genes present in the genome are nearly identical in sequence. Moreover, single-nucleotide polymorphisms and indels that vary among the rRNA gene pool (Sims et al., 2021), and might thus be targets for specific noncoding RNA interactions, are distributed throughout the rRNA gene repeats and do not cluster within known or suspected regulatory elements. Thus, we remain at a loss to understand how regulatory small RNAs might discriminate among rRNA genes in *A. thaliana*.

Our longstanding assumption that rRNA gene regulation occurs one gene at a time came to an end in our lab in 2016 when genetic evidence pointed to regulation on a larger scale (Chandrasekhara et al., 2016). Diploid *Arabidopsis thaliana* has two NORs that are located at the tops of chromosomes 2 (*NOR2*) and 4 *(NOR4*), with rRNA gene sequences fused directly to telomere repeats (Copenhaver et al., 1995, Copenhaver and Pikaard, 1996a, Mohannath et al., 2016). In the commonly studied *A. thaliana* accessions Col-0 and Ler, each NOR was estimated to have approximately 375 rRNA genes and span ~4 Mbp based on physical mapping studies using Contour-clamped Homogeneous Electric Field (CHEF) electrophoresis (Copenhaver and Pikaard, 1996b). As in other eukaryotes, the rRNA genes within an accession are nearly identical in sequence complexity. However, we identified a small set of single nucleotide polymorphisms or indels that differ between gene types that are developmentally silenced or are constitutively expressed in Col-0 (Chandrasekhara et al., 2016). Fortunately, natural variation for these molecular markers exists among different accessions of *A. thaliana*, and this allowed us to devise a genetic mapping strategy in which we simply monitored the segregation of the polymorphic rRNA gene markers relative to other chromosomal markers among the progeny of inter-accession hybrids. These genetic experiments revealed that the markers associated with active Col-0 rRNA gene subtypes mapped to *NOR4* whereas the silenced rRNA gene subtypes mapped to *NOR2* (Chandrasekhara et al., 2016)(see Figure 1d). This suggested that rRNA gene on/off fate might be determined by the NOR or chromosome in which the genes are located, consistent with many decades of cytological observations of NOR behavior, and not by individual rRNA gene sequences. Experimental support for this hypothesis came from our identification of a plant line in which a substantial portion of *NOR4* was replaced by sequences of *NOR2*. In this line, we found that the relocated rRNA genes of *NOR2,* which were normally silent in leaves, were now active (Mohannath et al., 2016). Collectively, these studies provided genetic evidence that the “choice mechanism” that determines which rRNA genes are silenced or active operates on a sub-chromosomal, megabase scale, possibly encompassing entire NORs. This hypothesis is compatible with a previous finding that rRNA transgenes inserted at genome positions outside the NORs escape silencing in hybrids in which the endogenous rRNA genes of the same sequence, located within the NORs, are subjected to nucleolar dominance and are silenced (Lewis et al., 2007). Likewise, NOR structural changes reflected by diminished rRNA gene numbers and altered subtype compositions occur in *fas* mutants (a subunit of CHROMATIN ASSEMBLY FACTOR 1) and disrupt the silencing of rRNA gene subtypes that normally map to *NOR2* (Pontvianne et al., 2013, Pavlistova et al., 2016), as do CRISPR-mediated deletions that dramatically reduce rRNA gene copy numbers and alter NOR organization (Lopez et al., 2021).

Interestingly, *NOR2* is not always silenced and *NOR4* is not always active in *A. thaliana*. In some accessions, rRNA genes that map to *NOR2* are active and genes mapping to *NOR4* are inactive; in other accessions, rRNA genes of both NORs are active (Rabanal et al., 2017). Cytogenetic studies in other species have also provided evidence that NOR activity can vary based on chromosomal and genomic context. For instance, in the cereal crop Triticale, which is the hybrid of wheat and rye, the wheat NORs are dominant and the rye NOR is suppressed (Thomas and Kaltsikes, 1983) (Lacadena et al., 1984) (Viera et al., 1990a, Viera et al., 1990b) (Silva et al., 1995) (Neves et al., 1997a, Neves et al., 1997b) (Amado et al., 1997). However, in a line in which a chromosome translocation event fused the NOR-bearing rye chromosome arm to a wheat chromosome, the rye NOR was shown to be active (Viera et al., 1990b). Studies of barley chromosome translocation lines also showed that rearrangements that move NORs to new chromosomal locations, or that delete sequences adjacent to NORs, can alter the on or off states of its NORs (Nicoloff et al., 1979, Schubert and Kunzel, 1990). Likewise, studies of nucleolar dominance in Drosophila, both in interspecific hybrids and pure species, have provided evidence that sequences flanking the NORs play a role in selective rRNA gene silencing (Durica and Krider, 1978, Warsinger-Pepe et al., 2020). In the case of *Arabidopsis thaliana*, strain Col-0, the NOR bearing the rRNA gene subtypes that are silenced, *NOR2*, is flanked on its centromere-proximal side by an approximately 70 kb region composed of transposable elements and transposon-derived sequences. By contrast, *NOR4* is flanked by protein coding genes, with the first gene located only ~3 kb from the NOR. Whether the transposon-rich flanking region plays a role in the developmental silencing of *NOR2* remains unknown and awaits direct testing, potentially by deleting the region through CRISPR-mediated chromosome engineering, mutagenesis and break repair, or genetic recombination. Complete end-to-end sequences of Arabidopsis *NOR2* and *NOR4* are also needed, to determine if clues to their differential activity might be hidden within the NORs. However, recent sequencing of bacterial artificial chromosome constructs bearing multiple rRNA gene subtypes that genetically map to *NOR2* have not revealed sequences other than rRNA gene repeats (Sims et al., 2021).

## The search for the off switch and its circuitry

Cytogenetic, genetic and molecular evidence implicate NORs as the likely units of large-scale rRNA gene regulation, with additional levels of regulation determining how many genes within an active NOR are transcribed, and at what levels. But what distinguishes one NOR from another? Are there sequences comprising a locus control element of some sort within, or adjacent to, NORs subjected to silencing but missing from NORs that are not silenced? If so, what is its composition and how does it work? Do these putative locus control element sequences become rearranged in *fas* mutants, thereby resulting in alternative rRNA gene subtype expression patterns (Pavlistova et al., 2016)? And what accounts for the fact that in a pure species such as *Arabidopsis thaliana*, where one NOR can be active and the other silenced, or both NORs can be co-dominant (depending on the accession), these dominance relationships become irrelevant when the genomes of *A. thaliana* and *A. arenosa* are combined to form the allotetraploid hybrid, *A. suecica* and both *A. thaliana*-derived NORs are silenced? What are the signals that initiate the silencing process depending on genomic context? Though answers to these questions are not yet in hand, the allure of science lies in the satisfaction of knowing that our reach can exceed our grasp.

## Significance statement

Nucleolar dominance is one the earliest recognized epigenetic phenomena, describing the expression of some, but not all, of the rRNA genes clustered in the nucleolus organizer regions of eukaryotic genomes. In the past five decades our conception of nucleolar dominance has evolved from models of selective gene activation to models of selective gene silencing, and from mechanisms thought to act on individual rRNA genes to mechanisms that may act on a multi-megabase scale.
